# The Blade of the *Western Lancet*

**Published:** 1878-02

**Authors:** 


### THE BLADE OF THE WESTERN LANCET.

The *Western Lancet* of last month expresses, in a passionate and personal editorial, its anger at the discovery, in our December issue, of an unfavorable review of Prof. Toland's Lectures on Surgery. It is claimed that the Chicago author was unfair, malicious and untruthful, and that he has shown that he was not a gentleman.

We beg our readers not to conclude, from the fact of our reference to this exhibition of feeling on the part of our little contemporary, that we are about to engage in a war of words with it, or with any other publication. The JOURNAL AND EXAMINER will never condescend to personalities of this sort. The JOURNAL AND EXAMINER is not at present attempting to rival the style of the "*Eatanswill Gazette.*" We are merely glad of the occasion, thus presented, for explaining our position with reference to the general subject of reviews and reviewers.

The editors of this journal do not hold themselves responsible for the views expressed either by their contributors or reviewers. But they have a duty to perform both to their subscribers and readers, their critics, and the authors whose works are submitted to them for comment. This duty the editors solemnly pledge themselves to perform, in the most full, fearless and impartial manner possible. When this cannot be done, and the review department of this journal degenerates into a collection of book-publishers' notices, we propose to resign the management into the hands of others.

To our readers and subscribers, we intend to present the most faithful criticism of each publication that it is possible for us to obtain, praising all real excellencies, denouncing everything false, superficial and worthless.

We shall also interpose to save our reviewers from all improper criticism. We can assure the *Western Lancet* that our books are distributed to none save those whom we know to be gentlemen of good standing and professional responsibility, capable of impartial and just criticism. Our readers are interested in seeing the reports of such men, but they do not in the least care to read what others think of such reports. We therefore decline all criticisms of critics, drawing the line at this point in order to avoid a descent into the devious paths of personal journalism.

To return to the particular review of which the *Lancet* complains, we admit at the outset that in proof-reading an error occurred by which the word *strength* was misquoted, so as to read *length*. For this, we are glad to apologize, and express our regret. In every other allusion, if the precise words of the author have not been quoted, his ideas have been reproduced in unequivocal terms. We have never as yet succeeded any better than our contemporaries, in having the *ipsissima verba* of each monthly issue absolutely correct. But the *Lancet*, in its hot zeal for Prof. Toland, claims that the error admitted was a willful perversion and misquotation, and that this is simply one of many other instances, in the same review.

We deny in the most emphatic manner that our critic was guilty of the very grave offense here charged. In order to establish the fact, it is merely necessary to return to the book itself and some of its other reviewers. A short time since we received by mail an extract from one of the San Francisco newspapers, containing a column or two of the most fulsome adulation of Prof. Toland and his book, ostensibly written by a layman. We do not know whether this was mailed to us by the editor of the *Lancet* or some other of Prof. Toland's friends and admirers, but we know that this is not the source from which American medical men generally receive their impressions of similar books.

Certainly few would dare to charge that the reviewer of the same volume for the *American Journal of the Medical Sciences*

of January, 1878, had been guilty of the identical unfairness, untruthfulness and willful misrepresentation which is laid at the door of our critic. And yet the opinions of the two gentlemen in Chicago and Philadelphia are singularly similar. According to the latter, the teaching of the book under consideration is "meagre to starvation;" no mention is made in it of atropia in the treatment of syphilitic iritis; pyæmia and erysipelas are not discussed; antiseptic surgery ignored; Buck's extension apparatus, as well as Smith's and Hodgen's splints, not mentioned; Esmarch's method, Pacquelin's knife and the galvano-caustic appliances not referred to; cases are introduced rather on account of the prominence of the patient than of the nature of the accident or disease. The author is charged with egotism and ostentatiousness; and the critic concludes by admitting his failure to find any reason for the publication of the work.

If the editor of the *Western Lancet* had read the review which immediately preceded that which gave him such offense, he would have seen that even the Transactions of the Illinois State Medical Society, which might have looked for the most flattering of encomiums in the home of its best friends, received at the hands of a reviewer no greater favor than did Prof. Toland's lectures.

We believe that we have said enough to refute the charge of willful misrepresentation in this case. We have no question as to Prof. Toland's personal and professional worth; it is his book which we cannot recommend to medical purchasers who read the columns of this journal. We think that the evidence justifies such a decision. But we are fully aware of the further fact, that neither the medical journals of the Pacific Coast, nor of Chicago, nor of Philadelphia, can decide the fate of the volume. It will stand or fall by its own merits or demerits; and time will surely pronounce sentence upon it.

---

ONE of the worst instances of depravity on record (we refer to the *New York Medical Record* of Dec. 22, 1877), is announced in that periodical in the following terms: "A surgeon was recently indicted at the Northampton Assizes (England), for rape *while having a tooth extracted!*"

